# Brain Innate Immune Response in Diet-Induced Obesity as a Paradigm for Metabolic Influence on Inflammatory Signaling

**DOI:** 10.3389/fnins.2019.00342

**Published:** 2019-04-24

**Authors:** Felipe Macedo, Lucas Souza dos Santos, Isaias Glezer, Fernanda Marques da Cunha

**Affiliations:** Departamento de Bioquímica, Escola Paulista de Medicina, Universidade Federal de São Paulo, São Paulo, Brazil

**Keywords:** hypothalamus, inflammation, metabolism, microglia, obesity, saturated fatty acids

## Abstract

Obesity is a predisposing factor for numerous morbidities, including those affecting the central nervous system. Hypothalamic inflammation is a hallmark of obesity and is believed to participate in the onset and progression of the obese phenotype, by promoting changes in neuronal functions involved in the control of metabolism. The activation of brain immune cells in the hypothalamus, which are represented by microglia and brain macrophages, is associated with obesity and has been the focus of intense research. Despite the significant body of knowledge gathered on this topic, obesity-induced metabolic changes in brain cells involved in innate immune responses are still poorly characterized due, at least in part, to limitations in the existing experimental methods. Since the metabolic state influences immune responses of microglia and other myeloid cells, the understanding and characterization of the effects of cellular metabolism on the functions of these cells, and their impact on brain integrity, are crucial for the development of efficient therapeutic interventions for individuals exposed to a long-term high fat diet (HFD). Here we review and speculate on the cellular basis that may underlie the observed changes in the reactivity and metabolism of the innate immune cells of the brain in diet-induced obesity (DIO), and discuss important points that deserve further investigation.

## Introduction

According to the World Health Organization, obesity has almost tripled since 1975 and is now considered a worldwide epidemic, affecting more than 650 million people^1^. This rapid increase in the number of obese individuals raises profound concerns in the health community, since obesity is a predisposing factor for a wealth of morbidities, many of which are life-threatening (Guh et al., 2009). In fact, obese people have been shown to be more prone to developing central pathologies, including: stroke, depression, and Alzheimer’s disease (Bruce-Keller et al., 2009).

One of the hallmarks of obesity is the presence of metaflammation, an atypical systemic and sterile inflammation. This inflammatory state involves the participation of the innate and adaptive immune systems, resulting in the production of cytokines, chemokines and inflammatory lipids (Iyer et al., 2010; Cooke et al., 2016; Ralston et al., 2017). Metaflammation has been detected in a variety of different tissues, including different areas of the brain (Beilharz et al., 2016; Guillemot-Legris et al., 2016).

Inflammation in brain areas, other than the hypothalamus, might certainly contribute to the onset and progression of some of the central pathologies associated with obesity, however, hypothalamic inflammation was previously shown to play a central role in disease processes, affecting the control of energy intake and energy expenditure (Velloso and Schwartz, 2011). Moreover, these inflammation-induced alterations in the mediobasal hypothalamus (MBH) directly impact the integrity of homeostatic processes, such as hepatic glucose storage and delivery into the blood (Mravec et al., 2018). Interestingly, some brain-related obesity effects are modulated by sex (reviewed in Morselli et al., 2016 and Ávalos et al., 2018). Since *in vivo* experiments tend to employ male animals, this must be taken into consideration when interpreting data obtained from animal studies.

Here we review a variety of factors that contribute to modifications of brain innate immune cell metabolism and reactivity in diet-induced obesity (DIO). In this particular context, it was found that very little is known about the metabolism of microglia and brain macrophages. Thus, since a high fat diet (HFD) impacts pro-inflammatory gene expression, activity and morphology of hypothalamic immune cells, future studies should focus on and investigate which metabolic pathways in these cells are modulated by DIO, and determine how these changes contribute to the progression of obesity.

## Diet-Induced Obesity (Dio) Leads to Hypothalamic Inflammation

In 2005, the first report showing evidence demonstrating an association between DIO and hypothalamic inflammation was published (De Souza et al., 2005). Since then, numerous groups have confirmed and extended this finding in both rodents and humans (Thaler et al., 2012; Schur et al., 2015; Valdearcos et al., 2015). It is also well established that a HFD rapidly induces hypothalamic inflammation, with an associated increase in inflammatory gene expression and gliosis, that subsides and returns if the HFD is not interrupted (Thaler et al., 2012; Berkseth et al., 2014). Interestingly, early hypothalamic inflammation can be observed weeks before adipose tissue (AT) expansion and inflammation (Thaler et al., 2012; Gao et al., 2014), suggesting that hypothalamic inflammatory signaling contributes to the genesis of the overt obese phenotype, and is not simply a consequence of peripheral inflammation. The central role hypothalamic inflammation plays in obesity was further supported by two studies reporting that the detrimental HDF-related effects could be alleviated through genetic ablation or pharmacological inhibition of hypothalamic inhibitor of NF-κB^2^ kinase subunit β (IKKβ) (Zhang et al., 2008; Posey et al., 2009). Although pro-inflammatory signaling in the hypothalamus is a key event in the onset of DIO, the widespread inflammation and metabolic changes promoted by a HFD may further impact the hypothalamus.

### A Different Inflammatory Profile in Obesity

Obesity is characterized by a distinct level of systemic innate immune response, often referred to as “chronic low grade inflammation.” Metabolic dysfunction is accompanied by increased levels of non-esterified fatty acids and systemic inflammatory mediators, such as plasma pro-inflammatory cytokines (Iyer et al., 2010). While a discussion on the accurate use of the term inflammation, for a systemic and chronic response, is beyond the scope of this review, the notion of a generalized response from tissues and blood immune cells, as well vascular endothelial cells, is quite commonsense these days. Interestingly, the presence of inflammatory molecules in the blood and CNS induces sickness behavior, as characterized by decreased food intake (reviewed in Thaler et al., 2010). The paradox on how DIO-mediated inflammation results in a different outcome has been discussed (Thaler et al., 2010). According to the authors, it is plausible that the net impact, resulting from the complex cellular interplay between hypothalamic cells, along with factors such as stimulus quality, duration and intensity, could account for these differences. However, it is clear that HFD-induced hypothalamic inflammation diverges from other pro-inflammatory stimuli that promote sickness behavior. Understanding how this diet triggers inflammation and identifying why innate immune cells in the hypothalamus respond differently is essential to combating obesity.

### The Unknown Pathway That Triggers Inflammation in the MBH

**FIGURE 1:**
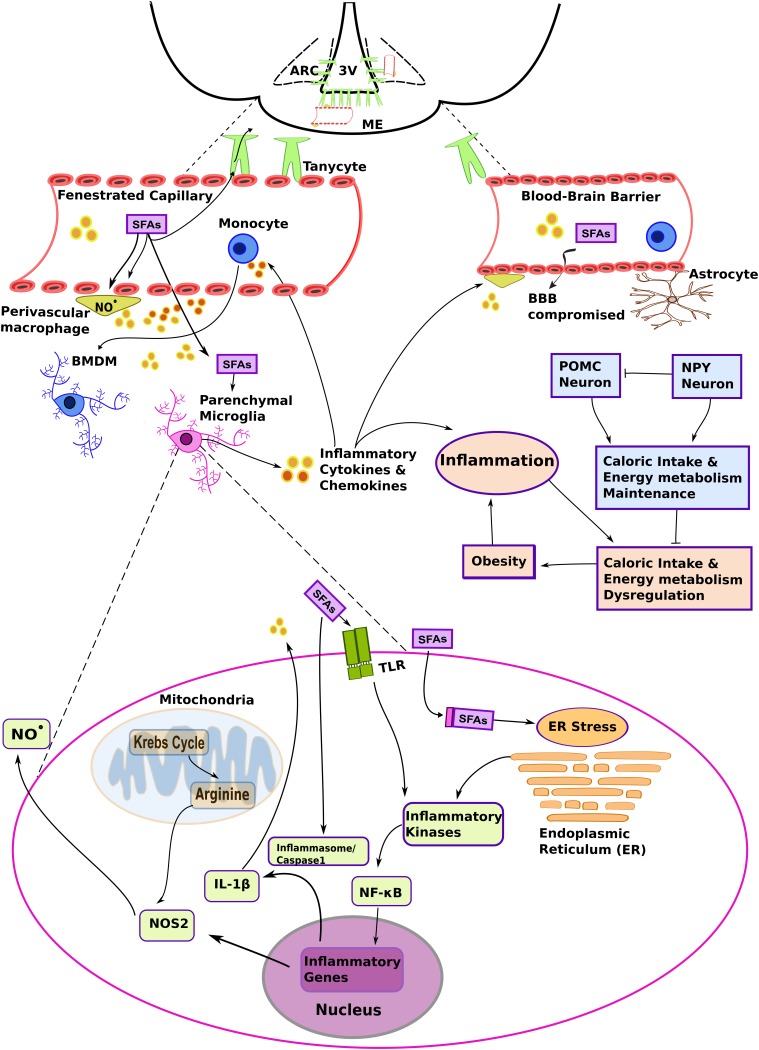
The complex interplay among different myeloid cells and metabolic substrates promotes hypothalamic inflammation in diet-induced obesity (DIO). A high fat diet (HFD) increases the levels of non-esterified saturated fatty acids in the blood and systemic inflammatory mediators, such as plasma pro-inflammatory cytokines. These molecules primarily target fenestrated capillaries in the median eminence (ME), and also influence the mediobasal hypothalamus (MBH) region, including the arcuate nucleus (ARC). Saturated fatty acids (SFAs) and inflammatory molecules activate brain endothelia and its associated immune cells, such as the perivascular macrophages. Changes in vascular permeability due to hypothalamic inflammation and signaling molecules such as nitric oxide (NO^⋅^), act in concert to promote lipid load accumulation in the MBH. Brain myeloid cells are heterogeneous and present particular phenotypes that result from the microenvironment and ontogeny. Resident parenchymal microglia are activated in DIO. Although several mechanisms still remain to be determined, it has been suggested that chronic low-level microglial activation perturbs metabolic control, and that this contributes to the development of the obese phenotype. At the same time, SFAs may influence the metabolic program and inflammatory signaling in microglia. The figure depicts hypothetical metabolic and inflammatory signaling changes in microglia based on data collected from metabolic macrophages (MMe) (see main text for details). Other abbreviations: 3V, third ventricle; BBB, blood brain barrier; BMDM, bone marrow-derived microglia; IL-1β, interleukin 1β. NF-κB, nuclear factor κB; NOS2, nitric oxide synthase 2 (inducible form; iNOS); TLR, *Toll*-like receptor.

It is important to note that the typical neurovascular unit that forms the blood-brain barrier (BBB) (Obermeier et al., 2013) is not the only example of vascular organization in the hypothalamus. Structures devoid of blood-barriers, such as fenestrated median eminence (ME) capillaries, or conversely some of the more tightly sealed endothelia in the arcuate nucleus (ARC), are actually surrounded by cellular processes projected from the third ventricle tanycytes (Langlet, 2014). This atypical cellular disposition contributes to the peculiar properties of the MBH in terms of collecting metabolic information carried by the blood stream (Figure 1). As an example, circulating pro-inflammatory mediators and biomolecules characterized as pathogen- and damage/danger- associated molecular patterns (PAMPs and DAMPs, respectively) were shown to be readily detected by brain immune cells associated with structures that lack the BBB (Rivest et al., 2000; Mallard, 2012). PAMPs and DAMPS represent an important concept in immunology, since these molecules are recognized by germline-encoded non-clonal receptors, and are the primary triggers of innate immune cell responses. However, it remains to be firmly established how an HFD could initiate hypothalamic inflammation.

Studies aiming at the characterization of the inflammatory molecules responsible for HFD-induced hypothalamic inflammation identified fatty acids as determinant factors. Indeed, a key characteristic of obesity is increased systemic free fatty acids, which accumulate in diverse tissues, including the hypothalamus (Boden, 2008; Posey et al., 2009; Valdearcos et al., 2014). In fact, the hypothalamus is particularly prone to the accumulation of long chain saturated fatty acids (SFAs), such as palmitoyl-CoA, thus promoting hypothalamic inflammation in animals fed a HFD. Moreover, while there has been no evidence showing that increased caloric or unsaturated fatty acid intake influences hypothalamic inflammation (Huang et al., 2004; Posey et al., 2009; Valdearcos et al., 2014), the amount of evidence implementing SFAs in this response is growing. With regards to the effects associated with other lipids, including unsaturated fatty acids, the reader is referred to dedicated reviews (White and Marette, 2014; Hammad and Jones, 2017; Rogero and Calder, 2018). A plausible rationale for how SFAs and HFD promote pro-inflammatory signaling in microglia cells—key players in DIO progression—will be presented in the next sections.

## Microglia as Primary Targets in Dio

Of the various cell types that populate the MHB, microglia have been the most studied in terms of DIO-induced hypothalamic inflammation, perhaps due to the fact that they are considered the resident brain macrophages. Indeed, microglial reactivity and inflammatory molecule production were found to be increased in the hypothalamus of animals fed an HFD and obese humans (Thaler et al., 2012; Gao et al., 2014; Valdearcos et al., 2014).

### Microglia Cells Respond to HFD and Play an Important Role in DIO

The fact that manipulation of microglia interferes with DIO indirectly shows that an HFD provokes profound effects on these brain myeloid cells, resulting in drastic metabolic consequences. Experimental approaches employing alternative strategies have demonstrated that microglia play a fundamental role in DIO. For example, interventions that impair microglia response (i.e., depleting these cells, restraining their responsivity or inhibiting microglia proliferation) alleviate the DIO phenotype and blunt the HFD-induced inflammation *in vivo.* A similar beneficial effect of microglial inhibition was observed in cultured brain slices stimulated with palmitate (Valdearcos et al., 2014, 2017; André et al., 2017). In contrast, forcing microglial activation by cell-specific deletion of tumor necrosis factor α-induced protein 3 (TNFAIP3), an anti-inflammatory molecule and negative regulator of NF-κB, rapidly induced obesity in mice, even when animals were fed control diets (Valdearcos et al., 2017). While this latter study does not explain how an HFD activates microglia, it certainly implicates hypothalamic microglia as a key player in DIO. Interestingly, persistent secretion of TNF-α by microglia in the MHB of mice fed a high fat/high carbohydrate (HFHC) diet was reported to cause dysfunction in anorexigenic neurons that produce proopiomelanocortin (POMC) (Yi et al., 2017), a characteristic feature of obesity (Pinto et al., 2004; Horvath et al., 2010). Assuming that lipids participate in microglial pro-inflammatory signaling, we direct the reader to Table 1, which summarizes the findings supporting a pro-inflammatory effect of SFAs on the hypothalamus and microglia.

**Table 1:** Microglia activation induced by high fat diet or fatty acid treatment.

Experimental model	Treatment	Duration of treatment	Results	References
Hypothalamus	Normal diet (ND)	13 and 16 weeks	↑ TNFα	De Souza et al., 2005
	HFD		↑ IL-1β	
			↑ IL-6	
				
		16 weeks	↑ TNFα mRNA	
			↑ IL-1β mRNA	
Hypothalamus	ND	16 weeks	↑ TNFα	Milanski et al., 2009
	HFD		↑ IL-1β	
			↑ IL-6	
				
	Intracerebroventricular	3 days	↑ IL-6 and IL-6 mRNA	
	infusion of oleic acid		↑ IL-10 and IL-10 mRNA	
				
	Intracerebroventricular	3 days	↑ TNFα	
	infusion of stearic acid		↑ IL-1β	
			↑ IL-10	
				
	Intracerebroventricular	3 days	↑ IL-6	
	infusion of linolenic acid		↑ IL-10	
				
	Intracerebroventricular	3 days	↑ TNFα	
	infusion of arachidic acid		↑ IL-1β	
			↑ IL-6	
			↑ IL-10	
				
	Intracerebroventricular	3 days	↑ TNFα	
	infusion of behenic acid		↑ IL-1β	
			↑ IL-6	
Hypothalamus	Intracerebroventricular	20 min	↑ pIKKβ	Posey et al., 2009
	infusion of palmitate		↓ IκBα	
				
	LFD	7 weeks	↑ pIKKβ	
	HFD			
Primary glial cells	Control serum	48 h	↑ CD11b positive cells	Hsuchou et al., 2012
	DIO serum			
BV-2 microglial cells	Vehicle	24 h	↓ TNFα mRNA	Kappe et al., 2012
	0.125 mM Palmitate		↑ Chi3L3 mRNA	
			↑ Arg1 mRNA	
Hypothalamus	ND	4 weeks	↑ TNFα mRNA	Thaler et al., 2012
	HFD		↑ IL-6 mRNA	
BV-2 microglial cells	Vehicle	24 h	↑ Arg1 mRNA	Tracy et al., 2013
	0.125 mM Palmitate			
Primary microglial cells	Control serum	120 min	↑ Iba-1	Gao et al., 2014
	HFD serum		↑ TNFα	
			↑ IL-1β	
Hypothalamus	ND	4 and 16 weeks	↑ Iba-1 positive cells	Valdearcos et al., 2014
	HFD	4 weeks	↑ TNFα mRNA	
			↑ IL-6 mRNA	
			↑ IL-1β mRNA	
				
	Enteric gavage of	3 days	↑ TNFα mRNA	
	lauric acid		↑ IL-6 mRNA	
				
	Enteric gavage of	3 days	↑ TNFα mRNA	
	palmitic acid		↑ IL-6 mRNA	
			↑ IL-1β mRNA	
				
Primary microglial cells	Vehicle	24 h	↑ TNFα	
	0.1 mM lauric acid		↑ IL-6	
			↑ MCP-1	
				
	Vehicle	24 h	↑ TNFα	
	0.1 mM myristic acid		↑ IL-6	
			↑ MCP-1	
				
	Vehicle	24 h	↑ TNFα	
	0.1 mM palmitic acid		↑ IL-6	
				
	Vehicle	24 h	↑ TNFα	
	0.1 mM stearic acid		↑ IL-6	
			↑ MCP-1	
BV-2 microglial cells	Vehicle	12 h	↑ iNOS mRNA	Duffy et al., 2017
	0.1 mM Palmitate			
Hypothalamus	ND	4 weeks	↑ Iba-1	Valdearcos et al., 2017
	HFD		↓ P2Y12	
BV-2 microglial cells	Vehicle	48 h	↑ IL-1β	Yang et al., 2017
	0.3 mM Palmitate		↑ CD11b mRNA	
			↑ IL-6	
			↑ MCP-1	
				
Hypothalamus	Low fat diet (LFD)	8 weeks	↑ TNFα mRNA	
	HFD		↑ IL-1β mRNA	


### Experimental Limitations and Thoughtful Interpretations

While the above-mentioned set of data suggests a prominent role for microglia in the detrimental effects of DIO, it is appropriate to introduce some notes of caution. At first glance, brain myeloid cells are thought to be either pathogen eradicators, or instigators of neuronal dysfuntion and cell death in various neurodegenerative diseases. However, microglia are important for the proper shaping of the brain, and function as a neuroprotective component of the innate immune response, a concept strongly supported by experimental data (Wyss-Coray and Mucke, 2002; Glezer et al., 2007; ElAli and Rivest, 2016). Thus, while experimental results indicate that preventing hypothalamic myeloid cell responses improves the outcome of HFD-treated animals, the consequences of DIO on the unprotected brain for a long period of time are not known. Secondly, due to limitations in experimental tools, “microglia” was frequently used as a generic term for all brain myeloid cells (myeloid as lineage term, not to be confused with bone marrow origin) in the past. Now it is known that in addition to the prototypical parenchymal microglia, other myeloid cells are present in the brain, including the non-parenchymal meningeal, perivascular and choroid-plexus macrophages, and disease-associated monocytes (Prinz et al., 2017). In this sense, it is likely that inflammatory signaling in the hypothalamus is coordinated by a complex interplay between microglia/macrophages, endothelial cells and macroglia (astrocytes, oligodendrocytes and their progenitors). Thus, efforts to target specific pathways and/or microglia/brain macrophage subpopulations are valuable and necessary, but the interpretation of the results should be performed carefully and objectively.

Lastly, it is important to keep in mind that brain myeloid cells do not necessarily influence metabolism through stereotyped responses to noxious stimuli. Indeed, myeloid cell specific *Cx3cr1*-driven ablation of receptors for the “satiety” hormone leptin was shown to result in increased food intake and body weight, dystrophic and less phagocytic microglia, and altered neuronal circuitry in the MBH (Gao et al., 2018). However, this genetic strategy cannot provide a conclusion about the specific role for leptin signaling in microglia/brain macrophages, since this approach may affect myeloid cells from multiple tissues, a commonly encountered experimental limitation associated with attempting to target, regulate and/or delete a specific gene in microglia (Han et al., 2017). The results, nonetheless, suggest that these cell types are responsive to a vast array of signals. Interestingly, recent data demonstrated that *Cx3cr1*-driven microglia and monocyte ablation in rats disrupts the gustatory circuitry at the paraventricular nucleus of the hypothalamus, leading to anorexia and weight loss (De Luca et al., 2019). Strikingly, it was reported that mice fed an HFD and treated with an intracerebroventricular injection of AraC, to block cell proliferation induced by HFD, presented reduced food intake, weight gain and adiposity, when compared with mice fed the same diet, but without being administered AraC (André et al., 2017). Thus, it is tempting to speculate that the reported benefits of microglia and monocyte ablation/inhibition in the DIO setting is secondary to decreased food intake, and not solely due to a reduction in inflammation.

Although many of the studies cited here indicate that there is a relationship among the HFD-induced increase in SFAs, brain myeloid cells pro-inflammatory response and hypothalamic energy metabolism control, the precise mechanisms linking these events are not definitively established. While improved experimental models and detailed molecular approaches are still necessary in order to unify these pieces of evidence into a compelling and complete model, it is clear that an HFD severely impacts the function of microglia.

## Lipids Activate Microglia/Macrophages

Since long chain saturated fatty acids seem to be important for HFD-associated hypothalamic inflammation, *in vitro* studies have been employed, using palmitate as a stimulus, for elucidating the molecular pathways involved in the microglial response to HFD. However, mixed results were reported, ranging from anti-inflammatory responses (Tracy et al., 2013; Kim et al., 2018) to pro-inflammatory ones (Wang et al., 2012). This discrepancy may stem, at least in part, from different lipid loads, since they may be modulated not only by the concentration of lipid used in the assays, but also by the ratio of lipid to the carrier protein albumin (Alsabeeh et al., 2018). Not surprisingly, the mechanisms through which parenchymal microglia respond to an HFD or SFAs are not clear yet, and much is still unknown. It is noteworthy that most of the research in the field has focused on typical macrophage readouts such as cytokine secretion, cellular recruitment, gene expression and cell morphology when exploring the effects of HFD or SFAs on microglia. Furthermore, data related to the mechanisms involved in the parenchymal microglia response to HFD or SFAs are extremely scarce. Therefore, we will summarize the findings related to macrophages, for which more information is available and discuss the extent to which these findings also apply to microglia.

### Macrophages and Microglia Do Not Necessarily Respond to HFD in the Same Way

With regards to macrophages, the response to SFAs seems to involve canonical and non-canonical mechanisms. The innate immune signaling is largely dependent on pattern recognition receptors, such as the *toll*-like receptors (TLRs). This family of membrane proteins activates pro-inflammatory transcription upon the recognition of structurally distinct PAMPs, such as lipopolysaccharide (LPS) from gram-negative bacteria (Takeuchi and Akira, 2010). Additionally, this response is complemented by intracellular protein complexes called inflammasomes, which sense PAMPs/DAMPs or relay upstream signaling, and promote the secretion of cytokines such as interleukin (IL)-1β (Sharma and Kanneganti, 2016). While SFAs are far from being considered PAMPs or DAMPs, similar response mechanisms have been described. For example, palmitate, but not unsaturated oleate, has been shown to activate the NLRP3-ASC inflammasome (Wen et al., 2011). Additionally, other studies also point to engagement of TLR4, the same receptor for LPS, by SFAs (Lee et al., 2001; Shi et al., 2006; Huang et al., 2012), suggesting that SFAs present the natural capacity to trigger innate immune cells. However, the evolutionary basis for the controversial idea that the immune system would respond against important caloric nutrients remains to be clarified.

How do the findings reported in macrophages apply to microglia? While it is expected that signals triggered by PAMPs and DAMPs follow quite similar pathways in different myeloid cells, it is important to mention that resident brain cells present unique and remarkable features. Molecular tracing and fate mapping studies revealed that microglia originate from yolk sac erythro-myeloid progenitors (primitive macrophages). Additionally, unlike macrophages from other tissues (that are largely derived from fetal monocytes), microglia and brain macrophages preserve their primitive lineage by means of self-renewal of resident progenitors, which infiltrated the brain during embryo development (Ginhoux et al., 2010; ElAli and Rivest, 2016; Prinz et al., 2017; Hoeffel and Ginhoux, 2018). This is relevant because microglia and brain macrophages maintain distinct repertoires of signaling and function, and long-term brain exposure to macrophage activating molecules will follow different cellular dynamics when compared to other tissues that actively replenish macrophages with bone marrow-derived cells. Furthermore, it is important to point out that environmental factors clearly determine the transcriptional programs that define the identity of microglia. For example, specific transcripts from murine microglia are lost when these cells are removed from the brain and transferred to cell culture (Gosselin et al., 2017). In addition, microglia completely regain their identity when transferred from *in vitro* culture to the brain *in vivo*, an event specific for yolk-sac primitive ontogeny, that cannot be replicated by engrafted macrophages from other tissues (Bennett et al., 2018). The authors of this study identified that the microglia-like cells, and not microglia, have a few unique markers that are found in pathological states of the human brain. The impact of these findings is very clear in terms of how crucial it is to determine which specific brain myeloid cells respond to HFD and SFAs, and the extent to which *in vitro* generated data can be translated to the hypothalamic niche of animals subjected to DIO.

Very recently, perivascular macrophages of the ARC, whose ontogeny has yet to be defined, were shown to proliferate *in situ* and express high levels of inducible nitric oxide synthase (iNOS/NOS2) when mice were fed an HFD (Lee et al., 2018). The same study also showed that hypothalamic NOS2 inhibition diminished several deleterious effects associated with DIO, including BBB permeability and lipid efflux (Lee et al., 2018). The idea that inflammatory cells regulate lipid uptake into the brain is quite attractive, but it is not clear how lipid overload, that occurs with an HFD, upregulates the expression of iNOS and other genes typically involved in pathogen defense. This is of particular relevance, since “chronic low grade inflammation” is commonly observed in obesity.

Due to the emergent nature of the subject, detailed studies describing the ontogeny and neuroanatomical details of the microglia/macrophages that are involved in the *in vivo* response to HFD, as well as studies combining specific gene promoters to drive pro-inflammatory signaling interference without disrupting brain barriers, are awaited. A comprehensive understanding of the molecular and cellular mechanisms involved in the response of microglia/brain macrophage to DIO and SFAs will likely reveal novel therapeutic targets for the prevention and/or treatment of obesity.

## Distinct Metabolic Programs Support Inflammatory Phenotypes in Macrophages and (Possibly) Microglia

It is now clear that immune cells have a profound impact on metabolic homeostasis and, that metabolic pathways can also significantly influence immune cell function. Indeed, the transition of immune cells from one phenotype to another is supported by and dependent on specific metabolic programs (reviewed in Van den Bossche et al., 2017). For example, macrophages stimulated with LPS(+ IFNγ) adopt a metabolic program that includes increased flux through glycolysis and the pentose phosphate pathway together with a reduction in oxidative phosphorylation (Rodríguez-Prados et al., 2010), due to a disruption of the citric acid cycle (Jha et al., 2015). This disruption repurposes the intermediary metabolites of the citric acid cycle for specific biosynthetic reactions, which sustain the inflammatory activities of LPS-stimulated macrophages (Tannahill et al., 2013; Littlewood-Evans et al., 2016; Mills et al., 2016; Williams and O’Neill, 2018). Interestingly, inhibition of specific mitochondrial membrane carriers impairs this repurposing and attenuates pro-inflammatory macrophage polarization by LPS, without affecting cell viability (Jha et al., 2015). Thus, indicating that metabolic pathways can influence the phenotype of immune cells and that modulating these pathways may represent a potent therapeutic strategy.

In obesity, macrophages acquire a specific phenotype that is strikingly different from the pro-inflammatory M1 phenotype, elicited by stimulation with LPS(+ IFNγ), and the anti-inflammatory M2 phenotype, induced by IL-4 (Lumeng et al., 2007; Zeyda et al., 2007; Kratz et al., 2014; Coats et al., 2017). In fact, macrophages stimulated with metabolic cues such as high SFAs, insulin and glucose, which are preponderant in obesity, are designated as metabolic macrophages (MMe). The MMe phenotype is associated with the production of cytokines, but presents a different set of surface molecules, when compared with macrophages stimulated with LPS. Additionally, these differences were also detected when macrophages isolated from the AT of obese patients were compared with macrophages isolated from the lungs of patients with cystic fibrosis, a disease in which the patients experience recurrent bacterial infections of the airway (Kratz et al., 2014). To date, little is known about the metabolic landscape of MMe macrophages, but different lipid metabolism programs seem to be important for MMe macrophage polarization and/or activity. In fact, SFAs seem to be important drivers of MMe activation, since macrophages stimulated with SFAs produce IL-1β and express surface markers characteristic of MMe isolated from obese mice (Kratz et al., 2014). The expression of many of the MMe surface markers are under the control of the lipid-engaged receptor PPARγ, reinforcing the idea that lipid metabolism is intimately linked to MMe activation and/or function. Indeed, it was shown that upon internalization, SFAs insert into phospholipid bilayer, changing the degree of membrane saturation. This altered lipid composition induces endoplasmic reticulum stress, a characteristic feature of MMe, which ultimately leads to the production of IL-1β (Robblee et al., 2016). Interestingly, the pharmacological inhibition of PPARγ or genetic inhibition of mitochondrial fatty acid oxidation in macrophages stimulated with SFAs exacerbate inflammation, and PPARγ deletion in macrophages aggravates insulin resistance in mice fed a HFD (Odegaard et al., 2007; Kratz et al., 2014; Namgaladze et al., 2014). Recently, gene expression data indicated that genes involved in glycolysis, oxidative phosphorylation and those related to lactate production were increased in AT macrophages isolated from obese mice, when compared to those isolated from lean animals. This metabolic program seems to be specific for AT macrophages as the expression of genes involved in energy metabolism was unaltered by an HDF in peritoneal macrophages (Boutens et al., 2018), highlighting the importance of the niche for macrophage response. Despite the data gathered so far, much more work is needed before we obtain a clear comprehensive understanding of the different metabolic programs that promote MMe polarization and activity (Namgaladze and Brüne, 2016), and how different niches (i.e., different AT depots) and the origin of the cell influence these responses.

With respect to microglia metabolism and its impact on cell phenotype and function, very little is known. Studies using LPS(+ INFγ) to stimulate microglia in culture reported increased pro-inflammatory cytokine and lactate production, which was accompanied by increased flux through glycolysis and the pentose phosphate pathway and decreased mitochondrial oxygen consumption (Voloboueva et al., 2013; Gimeno-Bayón et al., 2014). Thus, suggesting that there are parallels between macrophages and microglia in terms of their metabolic response to LPS(+INFγ), at least *in vitro*. However, these similarities seem to be limited, since there is still no clear evidence that microglia are responsive to IL-4, revealing the inadequacy of using the bimodal M1/M2 designation to describe the microglial response (Ransohoff, 2016). With regards to microglial metabolism in obesity, it was recently reported that impairing lipid uptake, through the silencing of lipoprotein lipase (LPL), specifically in microglia, exacerbates weight gain and glucose intolerance in mice fed an HFHC diet (Gao et al., 2017). The same study also reported that the absence of LPL in the microglia of mutant mice impaired the cellular responses (i.e., increase in number, changes in morphology and phagocytic capacity) observed in control mice fed an HFHC diet. In fact, this impairment was accompanied by an increased number of dysmorphic mitochondria in the microglia, possibly due to extensive alterations of the lipidome of those cells (Gao et al., 2017). While the work by Gao et al. indicated that lipid metabolism may play a central role in microglia phenotype and/or function in obesity, data related to microglia immunometabolism, especially in the context of obesity, are virtually non-existent. Thus, future studies investigating the modulation of microglia metabolism and, in a broader view, of myeloid cells are urgently needed, since these areas appear to be a promising avenue for treating obesity. It is important to note that although *in vitro* assays are important for providing mechanistic insights, *in vivo* work is crucial for accurately determining the metabolic response of microglia to different stimuli, as the niche exerts a profound influence on the cellular response. It is likely that changes in myeloid cell metabolism promote different outcomes in the MHB. In the context of obesity, more studies are needed to fully understand the impact of myeloid metabolism on cell function and reactivity; however, the origin of the cell and the niche must be taken into account when attempting to elucidate these response mechanisms.

## Concluding Remarks and Future Directions

Although much has been done, very little is known about how microglia cell metabolism affects the function of these brain cells. Additionally, it is unclear whether microglial metabolism is rewired, due to obesity-associated metabolic cues *in vivo*, or how this could occur. Due to the fact that microglia play a central role in obesity, and since obesity has devastating effects on human health, we believe that the metabolic program promoting obesity-induced microglial activation must be understood in detail. It will also be interesting to evaluate whether other brain myeloid cells present different metabolic programs, and to investigate the role each type of cell plays in hypothalamic inflammation. For example, if SFAs and myeloid cell metabolism induce an atypical inflammatory state, would it be possible to metabolically reprogram the microglia and restore normal hypothalamic function during DIO? Moreover, could the anti-inflammatory or deactivated microglia strategies employed in rodents be utilized in humans? Answering these questions, as well as others will pave the way for the development of therapeutic interventions aimed at preventing the onset and progression of obesity, as well as its associated co-morbidities.

## Author Contributions

All authors listed have made a substantial, direct and intellectual contribution to the work, and approved it for publication.

## Conflict of Interest Statement

The authors declare that the research was conducted in the absence of any commercial or financial relationships that could be construed as a potential conflict of interest.

## Footnotes

1 http://www.who.int/en/news-room/fact-sheets/detail/obesity-and-overweight

2 Nuclear factor kappa-B, a master activator of pro-inflammatory gene expression.
